# Polysubstance use and its correlation with psychosocial and health risk behaviours among more than 95,000 Norwegian adolescents during the COVID-19 pandemic (January to May 2021): a latent profile analysis

**DOI:** 10.1016/j.lanepe.2023.100603

**Published:** 2023-03-09

**Authors:** Rubén Rodríguez-Cano, George Kypriotakis, Laura Cortés-García, Anders Bakken, Tilmann von Soest

**Affiliations:** a PROMENTA Research Center, Department of Psychology, University of Oslo, Oslo, Norway; b Department of Behavioral Science, The University of Texas MD Anderson Cancer Center, Houston, TX, USA; c Norwegian Social Research (NOVA), Oslo Metropolitan University, Oslo, Norway

**Keywords:** Adolescent, Polysubstance use, Cigarette, e-Cigarette, Snus, Alcohol drinking, Cannabis, Illicit drugs, Latent profile analysis, COVID-19-Related problems

## Abstract

**Background:**

Polysubstance use represents an adolescent health risk; however, large-scale studies investigating this issue during the COVID-19 pandemic are scarce. We aim to (i) characterise substance use profiles among adolescents and (ii) identify correlates of such substance use profiles.

**Methods:**

Norwegian nationwide survey data from 2021 were analysed using latent profile analysis. Participants were 97,429 adolescents aged 13–18. We assessed cigarette, e-cigarette and snus use, alcohol consumption, and cannabis and other illicit drug use. Correlates included psychosocial variables, health risk behaviours, and COVID-19-related problems.

**Findings:**

We identified three adolescent profiles; those who use no substances (*n* = 88,890; 91%); those who use snus and alcohol (*n* = 6546; 7%); and those who use multiple substances (i.e., polysubstance profile; *n* = 1993; 2%). Boys, older adolescents, adolescents with lower socio-economic status, and those reporting low levels of parental control, and higher parental alcohol use, mental health problems, pain-related variables, and other health risk behaviours were most likely to be in the polysubstance profile. Adolescents with social and mental health issues related to COVID-19 were more at risk of being in the polysubstance profile. Adolescents who use snus and alcohol showed similar patterns of risk factors, but on a somewhat lower level than those in the polysubstance profile.

**Interpretation:**

Adolescents who use multiple substances have an unhealthier lifestyle, are at a higher risk of experiencing psychosocial impairments, and report more problems related to the COVID-19 pandemic. Preventative strategies to reduce polysubstance use might help promote psychosocial well-being in adolescents across various life domains.

**Funding:**

This study was funded by two grants from the 10.13039/501100005416Research Council of Norway (project #: 288083 and 300816). The 10.13039/501100014232Norwegian Directorate of Health has funded the data collection. The Research Council of Norway and the Norwegian Directorate of Health have not had any role in study design, data collection, data analysis, interpretation, and writing of the report.

**Research in context**

**Evidence before this study**

We searched for the literature in Embase and Pubmed for research articles until September 2022, using the following keywords (“adolescent” OR “young” OR “young people” OR “teenagers”) AND (“polysubstance use” OR “polydrug use” OR “substance use” OR “substance co-use”) AND (“latent class analysis” OR “latent profile analysis”, “Finite mixture modeling”). Although we identified several studies, they primarily used samples with 3000 or less participants, included participants mostly from North America or used data that were collected more than a decade ago. These characteristics do not allow the identification of potential substance use profiles based on the contemporary trends of substance use among adolescents (e.g., the use of non-combustible nicotine products). Likewise, most studies solely analysed the relationship between polysubstance use profiles and some selected variables of adolescents' life (i.e., socio-demographics and mental health mainly). Thus, previous studies provide limited understanding of how specific polysubstance and substance use profiles are related to a large array of psychosocial and health characteristics of adolescents' life. Furthermore, using several polysubstance use correlates within the same large sample will help to better understand the impact of substance and polysubstance use on adolescents’ life. Studies also showed that the COVID-19 pandemic has increased mental health problems and changed substance use patterns. However, the extent to which substance use patterns are related to COVID-19 related problems has yet to be studied. Adolescence is a period when substance use usually starts. It is therefore critical to identify substance use profiles and their correlates among adolescents in order to reduce the negative impact of drug use in their life.

**Added value of this study**

Using latent profile analysis (LPA) in a sample size of more than 95,000 participants, we provide the largest and most contemporary analysis of substance use profiles among European adolescents to date. LPA is a person-oriented approach that classifies individuals into profiles according to characteristics (i.e., frequency of consumption). We followed the latest methodological recommendations in LPA by examining several variance-covariance structures and included post-hoc bootstrapping tests. We found three profiles of substance use among adolescents; those who use no substances (n = 88,890; 92%); those who use snus and alcohol (n = 6546; 6%); and those who use multiple substances (i.e., alcohol, cannabis, and other illegal drugs; n = 1993; 2%). In times of declining cigarette use among adolescents, our study highlights that alternative nicotine products, such as Swedish snus, along with alcohol use, have a key role in substance use profiles among today's adolescents. Furthermore, to better understand the impact of substance and polysubstance use on adolescent health, this is the first study that included a broad range of demographic, psychosocial and health variables such as age, gender, socioeconomic status, parental and peer-relational variables, mental health, conduct problems, pain-related variables, health risk factors, and COVID-19 related problems.

**Implications of all the available evidence**

The findings of this study provide information about contemporary substance use profiles and the association with adolescents' health. This information is valuable to health care professionals and prevention policy makers. Prevention and intervention efforts that focus on reduction of both nicotine products and alcohol might promote adolescents' mental and physical health. Importantly, the use of alcohol, cannabis as well as other illicit drugs are central among adolescents with increased levels of mental and physical health problems. Hence, preventive efforts that reduce the use of several of these drugs might improve adolescents' health. Moreover, the results underline that adolescents who use several drugs are more exposed to psychosocial problems associated with the COVID-19 pandemic. Therefore, prevention and intervention efforts aiming at alleviating adverse effects of the COVID-19 pandemic should also target polysubstance use among adolescents. The current study may also lay the groundwork for future personalised interventions and randomised controlled trials based on polysubstance use profiles of adolescents, by providing information about psychosocial and health consequences of adolescents’ polysubstance use.

## Introduction

Adolescent polysubstance use is associated with various negative outcomes, including an increased risk of developing physical and mental health problems.^1^^,^^2^ Polysubstance use is defined as the consumption of more than one drug simultaneously or at different times^3^ and has been studied repeatedly among adolescents.^4^, ^5^, ^6^ However, profiles of polysubstance use integrating new epidemiological consumption patterns, such as the reduction of traditional combustible cigarettes in favour of e-cigarettes, are underexplored.^6^ Specifically, in the Nordic context, where a non-combustible tobacco product known as snus is widely used, research on polysubstance use is scarce.^7^ To address this gap in the research, we apply latent profile analysis (LPA) to a large, nationwide dataset of Norwegian adolescents who were assessed during the COVID-19 pandemic in 2021 (N = 97,429) in to order to (i) characterise adolescent substance use profiles and (ii) identify correlates of substance use profiles from different arenas of adolescents’ lives.

LPA has been demonstrated to be a methodologically sound, person-oriented approach to identifying adolescent substance use profiles, with subgroups of individuals classified into profiles based on characteristics and consumption frequency.^5^^,^^8^^,^^9^ Studies using the LPA methodology differ considerably on how drug use has been assessed, both in terms of the number and types of drugs measured and in terms of whether recent drug use or drug use over longer periods or even the lifetime was assessed.^4^^,^^5^^,^^10^ Even though such measurement issues may potentially make comparison across studies difficult, most studies tend to provide surprisingly similar results, suggesting three to four latent groups for adolescents, independent of study design.^5^ The most commonly identified profiles are those of non-users or low-frequency users, followed by an alcohol user group and a smaller polysubstance user group.^5^ The most common polysubstance use groups include alcohol, tobacco, and cannabis use. Studies with four latent groups typically identify an additional polysubstance group, including the use of several substances, such as other illicit drugs than cannabis.^11^ Notably, most studies examining adolescent substance use profiles use sample sizes of 3000 or less, while few include larger samples. Applying LPA to data with a larger sample may increase the accuracy of the identified profiles and reduce profile misspecification.^12^ This issue may be particularly important when studying phenomena of low prevalence, such as polysubstance use among adolescents, where large-sized samples provide a sufficient number of adolescents for each profile.

One of the few studies with a comprehensively large sample size examined 51,767 Canadian adolescents and found polysubstance use to include alcohol, cannabis, cigarettes, and e-cigarettes use.^10^ In another large-scale study from Sweden, on a dataset of 20,057 adolescents, polysubstance use comprised alcohol and tobacco use, drunkenness and inhalant use.^4^ However, the data used in this study were over ten years old and consequently assessed neither e-cigarette nor snus use, which have become increasingly prevalent in Nordic countries during the past decade. Aside from this Swedish study, almost all large-scale studies use North American samples, and no studies have differentiated between the various tobacco products commonly used by Nordic adolescents.

The COVID-19 pandemic has considerably impacted adolescent lives and led to increasing mental health problems and changes in substance use patterns.^13^^,^^14^ However, the extent to which substance use patterns are related to COVID-19-related problems has yet to be studied, leaving an unfilled gap in the research literature. Conducting large-scale studies using current Nordic datasets that distinguish snus use from the use of other tobacco products and that account for adolescent substance use patterns during the COVID-19 pandemic will aid in understanding substance use profiles among the adolescents of today.

Previous LPA studies have analysed the relationship between several variables that are suggested to be related to adolescent polysubstance use (for an overview, see^5^^,^^9^). For example, older male adolescents and adolescents from lower socio-economic backgrounds are more likely to use several substances.^5^^,^^9^ Other studies show that low levels of parental monitoring,^15^ high incidences of mental health problems,^9^ high levels of conduct problems,^16^ and physical pain (i.e., headache and back pain) are associated with polysubstance use.^1^ Finally, although health risk behaviours, such as a poor diet and low levels of physical activity, have rarely been included in LPA polysubstance use studies, a few studies found that high levels of alcohol and tobacco use are related to the low consumption of fruit and vegetables^17^^,^^18^ and low levels of physical activity.^18^ Even though these studies found an overall relationship between these variables and polysubstance use, few studies have included a wide array of correlates using the same sample. By doing so in the present study, we may better understand how various factors in the health behaviours domain are related to polysubstance use among adolescents.

By analysing measures of use of cigarettes, e-cigarettes, snus, alcohol, cannabis, and other illicit drugs from a nationwide sample of Norwegian adolescents from 2021 (N = 97,429), this study aims (i) to identify substance use profiles and (ii) to examine the association of substance use profiles with socio-demographic variables, psychosocial variables (including relationships to parents and peers, mental health, and conduct problems), pain-related variables, health risk behaviours, and COVID-19-related problems. This study has several advantages compared to previous studies: We use one of the largest samples collated in the Nordic countries with current data on drug use prevalence; we apply an analytical approach that might reduce the problems with profile identification (i.e., LPA with different variance-covariance structures); and we include specific problems related to the COVID-19 pandemic and their relationship with substance use profiles. The study will provide knowledge that may help inform and tailor prevention and intervention efforts to reduce adolescent substance use.

## Methods

### Procedure and participants

We use data from the Norwegian nationally representative *Ungdata* surveys. *Ungdata* is a national data repository of youth surveys in Norwegian municipalities. All junior and senior high school students from grades 8 to 13 (aged 13–18) were invited to participate. Students and parents were informed that participation was voluntary, and the survey was completed in an electronic format in class.

For the present study, we included all students participating in *Ungdata* surveys in 2021. Data were collected from January to May when all students in 204 of Norway's 356 municipalities were asked to participate, and 101,779 of them agreed (77% response rate, 51% girls). We excluded data from 4350 participants due to missing values on all substance use indicators, resulting in a sample of 97,429 adolescents.

The University of Oslo's Department of Psychology internal research ethics committee approved the study (reference #13710027).

### Measures

#### Latent profile indicators of substance use

The use of cigarettes was assessed by one item with response options “I have never smoked” (coded 0), “I used to smoke, but I stopped completely now” (0), “I smoke less than once a week” (1), “I smoke every week, but not every day” (2), and “I smoke every day” (3). The first two response options were both coded 0, as both options indicated no current cigarette use. The use of e-cigarettes and snus was assessed by two additional questions and coded similarly. Alcohol use was measured by one item with response options ranging from 0, “Never”, to 4, “Every week”. Cannabis use and ‘other illicit drug use’ during the previous 12 months were measured with response options ranging from 0, “never”, to 4, “11 or more times”. See Table S1 for a detailed account of all substance use items and their response options.

#### Correlates

##### Demographics

Gender was assessed by self-report. Age was assessed indirectly by school grade, where grade 8 corresponds to age 13 and grade 13 to age 18. Attendance in school grades is strictly organised by birth cohorts in Norway, and failing a grade due to poor academic performance is uncommon.

##### Socio-economic status

We used the four-point Family Affluence Scale (FAS)^19^ to measure family economic status. We also asked about parental university education (“neither of them”, “one of them”, and “both of them”).

##### Social and relational variables

Parental control was assessed using four items with statements such as: “My parents usually know where I am, and who I am with, in my free time”. The students responded on a 4-point scale from 0, “very true”, to 4, “not true at all”. Parental permissiveness regarding alcohol use by their adolescent was assessed by asking, “Do your parents allow you to drink alcohol?” with the response options “no” (0) and “yes” (1). Adolescents also indicated if they were offered cannabis during the previous 12 months, coded as “no, never” (0), “yes, once” (1) and “yes, several times” (2).

##### Mental health

Depressive symptoms were assessed by the 6-item Depressive Mood Inventory developed by Kandel and Davies,^20^ with response options ranging from 1, “not affected at all”, to 4, “extremely affected”. The instrument showed a good internal consistency in the present study was α = 0.89. Loneliness was assessed by one item about feeling lonely during the previous week, with the same response options as for depressive symptoms. Visits to a clinical psychologist during the previous 12 months were also assessed (from 1, “never”, to 4, “6 or more times”).

##### Conduct problems

The prevalence of five behaviours was assessed to measure conduct problems during the previous 12 months (stealing, vandalism, tagging, truancy, accessing public transportation, or events without paying). Responses ranged from 1, “never”, to 5, “11 or more times”. Mean scores were computed, and the scale's internal consistency was α = 0.71.

##### Pain-related variables

We assessed the frequency of headaches and other pain (i.e., nausea, stomach-ache, and joint, neck, or muscle pain) with two items; response options ranged from 1, “never”, to 4, “daily”. Painkiller use (e.g., paracetamol) was also assessed, where response options ranged from 1, “never”, to 5, “daily”.

##### Health behaviours

Adolescents reported their frequency of fruit consumption per week (from 0, “non-frequent”, to 3, “5 days per week”). They also reported how frequently they engaged in physical activity that made them out of breath or sweaty (from 0, “never”, to 6, “at least 5 times per week”).

##### COVID-19-related problems

We assessed adolescents’ experiences during the pandemic compared to their pre-pandemic situation. Similarly to a previous study,^21^ three items were used to assess mental health (stress, worry, general mood), four items were used to assess the relationship with peers (talking, leisure time, social contact, and loneliness), and two items were used to assess the relationship with parents (arguments, spending fun time together). Response options ranged from 1, “strongly disagree”, to 4, “strongly agree”.

### Statistical analyses

To identify substance use profiles, we used LPA, where all six indicators of substance use (i.e., cigarette, e-cigarette, snus, alcohol, cannabis, and ‘other illicit drug’ use) were included as continuous variables. Following recent guidelines on applying LPA,^12^ we ran an iterative process to identify the best profile solutions and their correlates. 1) *Profile enumeration phase.* We tested four different variance-covariance structures for profiles (i.e., invariant diagonal, varying diagonal, invariant non-diagonal, and varying non-diagonal) for each set of models ranging from 1 to 6 profile solutions. We verified the replication of the best log-likelihood value to avoid local maxima with three different sets of random starting points among each model. 2) *Model evaluation.* We used the Bayesian information criterion (BIC) and sample size adjusted BIC (SABIC) to assess model fit. We also evaluated log-likelihood-based indices, such as the adjusted Lo-Mendell-Rubin likelihood ratio test (LMR-LRT) and the bootstrapped likelihood ratio test value (BLRT). Statistically significant results indicate that the *K profile* model fits the data better than the *K-1 profile* model. However, in studies with large sample sizes, the fit indices may have significant values in all comparisons, even when practical significance is low.^12^ 3) *Contender model assessment.* Once we identified the preferred model (i.e., the contender model), we analysed whether the profiles of the model were identified correctly by calculating the Average Posterior Probability (AvePP) and Odds of Correct Classification (OCC). AvePP closer to 1 and OCC >5 support adequate profile separation and precision. 4) *Latent profiles correlates.* Finally, we assessed correlates of substance use profiles from the contender model by including potential correlates as predictors of latent profiles in multinomial logistic regressions with the three-step approach in Mplus 8.5.^22^ We reported odds ratios (ORs) and ORs with adjustments for age, gender, socio-economic status, parental control, and parents' permissiveness with adolescents' alcohol use.

LPA analyses were run with Mplus 8.5.^23^ We set the level of significance to p < 0.01. We used full maximum likelihood estimation with robust standard errors to estimate the latent profiles. Moreover, multiple imputations with ten imputation samples were conducted to handle missing data in all logistic regression analyses under the missing at random (MAR) assumption.

### Role of funding

This study was funded by two grants from the Research Council of Norway (project #: 288083 and 300816). The Norwegian Directorate of Health has funded the data collection. The Research Council of Norway and the Norwegian Directorate of Health have not had any role in study design, data collection, data analysis, interpretation, and writing of the report.

## Results

Descriptive statistics and correlations among all six substance use indicators are displayed in Table 1 and Table S1. Overall, adolescents reported low means and frequencies for all variables. Alcohol use had the highest mean (M = 1.14, SD = 1.22). More specifically, 44% of all participants reported never having consumed alcohol, whereas 14% consumed alcohol quite regularly (1–3 times per month) and 4% every week (see Table S1). Regarding tobacco use, snus use was most frequently used, with 6% of all adolescents using snus every day, whereas 1% smoked cigarettes and 1% used e-cigarettes daily. Of all adolescents, 7% had used cannabis at least once, whereas 3% reported having used other illicit drugs at least once in the previous 12 months. All six drug use indicators were positively correlated, ranging from *r* = 0.22 for the association between alcohol and other illicit drug use to *r* = 0.52 for the association between cannabis and other illicit drug use. Descriptive statistics for all correlates are displayed in Table S2.

**Table 1:** Descriptive statistics and correlations of drug use variables.

Variable	*M*	*SD*	Median	Range	n	1	2	3	4	5
1. Cigarette use	0.14	0.48	0	[0–3]	96,800					
2. Snus use	0.26	0.78	0	[0–3]	96,769	0.55 [0.54, 0.55]				
3. E-cigarette use	0.07	0.38	0	[0–3]	96,474	0.40 [0.39, 0.41]	0.34 [0.33, 0.35]			
4. Alcohol use	1.14	1.22	1	[0–4]	96,643	0.41 [0.41, 0.42]	0.44 [0.43, 0.44]	0.23 [0.22, 0.24]		
5. Cannabis use	0.14	0.62	0	[0–4]	96,643	0.43 [0.42, 0.44]	0.37 [0.36, 0.37]	0.26 [0.25, 0.27]	0.32 [0.31, 0.33]	
6. Use of other illicit drugs	0.08	0.46	0	[0–4]	96,087	0.32 [0.31, 0.33]	0.28 [0.27, 0.29]	0.27 [0.26, 0.28]	0.22 [0.21, 0.23]	0.52 [0.51, 0.53]

*M* = mean; *SD* = standard deviation. Spearman rank-order correlations were used. All correlations were statistically significant at p < 0.001. Values in square brackets indicate the 99% confidence interval for each correlation.

### Latent profile analysis

In the profile enumeration phase, only two variance-covariance specifications converged for the complete range of models from one to six profiles; the profile-invariant diagonal specification (i.e., variances are constrained to be the same in each profile and residual covariances are not estimated), and the profile-invariant unrestricted specification (i.e., variances and residual covariances are estimated but constrained to be equal across profiles). Although both variance-covariance structures replicated their log-likelihood values, the percentages of replication of the profile-invariant diagonal specification were much higher across all models (Table 2). Thus, we evaluated the best model of profiles using the profile-invariant diagonal specification.

**Table 2:** Model fit indices from latent profile analysis of substance use among Norwegian adolescents (N = 97,429).

Variance-covariance structure	No. of profiles	LL	% of LL replication	Number of parameters	% of participants in the smallest profile	Information criteria		Likelihood ratio tests, p	
						BIC	SABIC	LMR-LRT	BLRT
Profile-invariant diagonal	1-profile	−530,857	100	12	100	1061851.60	1061813.47	–	–
	2-profile	−399,393	100	19	7.95	799044.49	798944.10	<0.0001	<0.0001
	3-profile	−322,487	100	26	2.05	645272.82	645190.19	<0.0001	<0.0001
	4-profile	−264,946	98	33	1.24	530270.78	530165.91	<0.0001	<0.0001
	5-profile	−204,921	18	40	0.94	410301.42	410174.30	<0.0001	<0.0001
	6-profile	−179,694	11	47	0.90	359927.88	359778.51	<0.0001	<0.0001
Profile-varying diagonal	1-profile	−530,857	100	12	100	699245.48	699137.42	–	–
	2-profile	−528,046	8	25	0.18	1056379.37	1056299.92	<0.0001	<0.0001
Profile-invariant non-diagonal	1-profile	−512,156	100	22	100	1024563.96	1024494.05	–	–
	2-profile	−349,428	100	34	7.92	699245.84	699137.78	<0.0001	<0.0001
	3-profile	−293,177	100	41	5.25	586825.53	586695.23	0.334	<0.0001
	4-profile	−239,556	26	48	1.84	479662.43	479509.89	<0.0001	<0.0001
	5-profile	−184,681	12	55	0.96	369994.00	369819.21	<0.0001	<0.0001
	6-profile	−169,116	1	62	0.70	338944.59	338747.56	<0.0001	<0.0001
Profile-varying non-diagonal	1-profile	−446,248	100	27	100	892807.09	892721.28	–	–

LL = loglikelihood; npar = number of parameters; BIC = Bayesian information criterion; SABIC = sample size adjusted BIC; LMR-LRT = p-value of the adjusted Lo–Mendell–Rubin likelihood ratio test; BLRT = p-value of the bootstrapped likelihood ratio test. Profiles not displayed did not converge or had problems in the estimation with models' parameters.

Fit indices provided inconclusive information about the number of profiles because they did not present a clear plateau and all the tests showed statistical significance (Table 2). Based on theoretical grounds to select the best model, the 4- and 3-profile models emerged as the best models because they showed interpretable profiles. However, the 4-profile model presented estimation problems with the variance for the ‘other illicit drugs’ indicator and reduced the smallest class to only 1% of the total sample. Thus, the 4-profile model did not provide a correct identification.^a^ Therefore, we finally selected the 3-profile model as the best model as it returned an appropriate profile separation and identification (AvePP ∼ 1 and OCCs > 5) (see Table 3). Moreover, we re-ran the 3-profile solution in the framework of latent class analyses where substance use indicators were treated as categorical indicators and found similar class/profile distributions than for the LPA 3-profile solution, thereby supporting the robustness of the identified profiles.

**Table 3:** Classification quality of final enumerated 3-profiles invariant diagonal model (N = 97,429).

Profiles	Model estimated class proportion		mcaP	AvePP	OCC
	Percentage	99% C.I.			
Non-users	91%	0.910–0.915	0.912	0.999	98.80
Snus and alcohol	7%	0.065–0.069	0.067	0.998	6629.57
Polysubstance	2%	0.019–0.022	0.021	0.997	16284.33

mcaP = modal class assignment Proportion; AvePP = Average Posterior Probabilities; OCC = Odds of Correct Classification, which is odds of model estimated class assignment relative to random assignment by class proportion. AvePP close to 1 and OCC > 5 support adequate profile separation and precision.

**Fig. 1:**
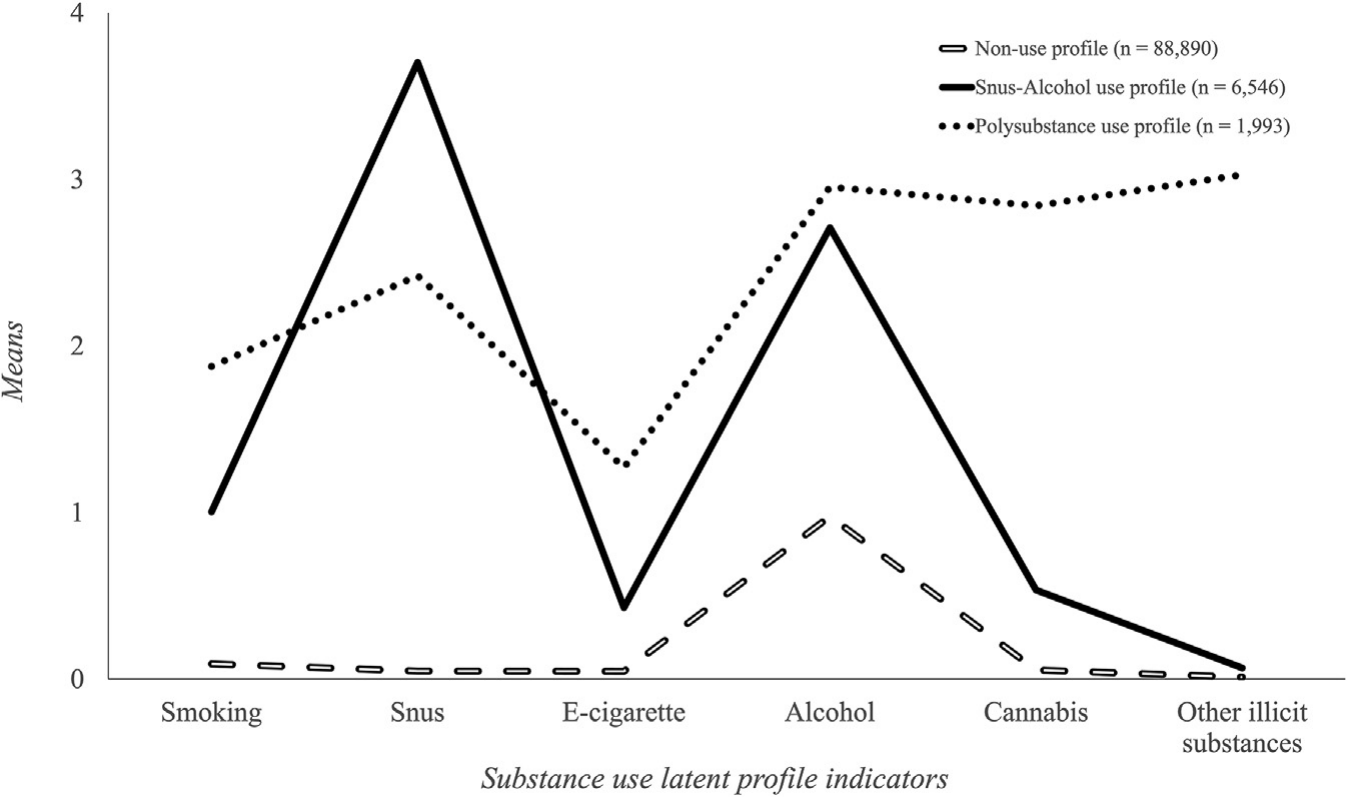
**Substance use indicators means by substance use profile**. The maximum possible scores for different substance use indicators ranged from 3 for nicotine products to 4 for other substances (alcohol, cannabis and other illicitly substances). To obtain a comparable scaling in the figure, we rescaled indicators for use of nicotine products, such that the maximum possible score was 4 for all variables (i.e., raw scores of nicotine variables were divided by 3 and then multiplied by 4).

Fig. 1 displays the three identified profiles. The first profile included adolescents who were *non-users* (*n* = 88,890, 91% of the total sample), with means close to 0 for all types of substance use, except alcohol, where the mean of 0.98 (*SD* = 1.11) indicates that adolescents in this profile had on average consumed alcohol a few times in their life. The second profile included adolescents who used s*nus and alcohol* (*n* = 6546, 7%). This profile was characterised by an average daily consumption of snus (*M* = 2.78, *SD* = 0.08) and an average consumption of alcohol of 1–3 times per month (*M* = 2.71, *SD* = 1.23), while consumption of other substances was low. The third profile consisted of adolescents who used several substances (*polysubstance use; n* = 1993, 2%). This profile included the highest means among all substances, except for snus use (*M* = 1.82, *SD* = 0.28), which was higher in the *snus and alcohol* profile. Substance use frequencies by profile are presented in Table S2.

### Latent profiles correlates

Next, we identified correlates of substance use profiles using multinomial logistic regressions. Descriptive statistics of the correlates by class are presented in Table S2. Table 4 shows the results by means of unadjusted odds ratio (OR) and adjusted OR (AOR). The OR can be interpreted as the change in odds of being a member of a specific profile compared to a reference profile with one unit change in the correlate. Older adolescents and boys were more likely than other adolescents to have a polysubstance use profile compared to non-users; age did not remain a significant correlate when controlling for covariates. Adolescents with lower scores on the family affluence scale were more likely to be in the polysubstance use profile compared to the non-user profile, while parental education was significantly correlated in some analyses.

**Table 4:** Results of multinomial logistic regression analyses of the associations between measures in various domains of adolescents’ lives and latent substance use profiles.

Correlates	Snus and alcohol use versus non-use^a^		Polysubstance use versus non-use^a^		Polysubstance use versus snus and alcohol use^a^	
	OR [99% CI]	AOR [99% CI]	OR [99% CI]	AOR [99% CI]	OR [99% CI]	AOR (99% CI)
**Demographics**						
Age	1.70 [1.67–1.74]	1.44 [1.40–1.48]	1.32 [1.27–1.38]	1.04 [0.99–1.09]	0.77 [0.74–0.81]	0.72 [0.68–0.76]
Female gender	0.62 [0.58–0.66]	0.55 [0.51–0.59]	0.45 [0.39–0.51]	0.43 [0.38–0.49]	0.73 [0.63–0.84]	0.79 [0.68–0.91]
**Socioeconomic status**						
Family affluence	0.65 [0.61–0.70]	0.82 [0.75–0.89]	0.33 [0.29–0.37]	0.46 [0.41–0.52]	0.50 [0.44–0.57]	0.56 [0.49–0.65]
Parental education	0.80 [0.78–0.83]	0.87 [0.85–0.90]	0.82 [0.78–0.86]	0.98 [0.93–1.03]	0.98 [0.92–1.03]	1.13 [1.06–1.19]
**Social and relational variables**						
Parental control	0.55 [0.51–0.60]	0.75 [0.69–0.81]	0.21 [0.19–0.25]	0.32 [0.28–0.37]	0.39 [0.34–0.45]	0.43 [0.37–0.51]
Parent's permissiveness towards adolescents' alcohol use	4.92 [4.60–5.26]	2.29 [2.09–2.51]	4.10 [3.63–4.64]	3.30 [2.77–3.95]	0.83 [0.73–0.96]	1.44 [1.19–1.76]
Having been offered cannabis	3.47 [3.34–3.60]	2.83 [2.71–2.95]	10.80 [9.75–11.97]	9.36 [8.41–10.41]	3.11 [2.80–3.46]	3.31 [2.96–3.70]
**Mental health**						
Depressive symptoms	1.43 [1.38–1.49]	1.52 [1.45–1.59]	2.53 [2.33–2.75]	2.59 [2.36–3.84]	1.76 [1.61–1.93]	1.70 [1.54–1.88]
Loneliness	1.08 [1.05–1.12]	1.09 [1.05–1.13]	1.65 [1.55–1.75]	1.54 [1.45–1.65]	1.53 [1.43–1.63]	1.42 [1.32–1.52]
Contact with clinical psychologist	1.32 [1.27–1.37]	1.35 [1.29–1.40]	1.86 [1.77–1.96]	1.87 [1.78–1.98]	1.41 [1.33–1.50]	1.39 [1.30–1.48]
**Conduct problems**	3.55 [3.39–3.72]	3.36 [3.19–3.55]	7.78 [7.26–8.35]	6.76 [6.26–7.30]	2.20 [2.06–2.34]	2.01 [2.87–2.16]
**Pain**						
Frequency of headaches	1.24 [1.19–1.29]	1.40 [1.33–1.47]	1.72 [1.59–1.85]	1.85 [1.70–2.01]	1.38 [1.27–1.50]	1.32 [1.21–1.45]
Frequency of other pain	1.16 [1.12–1.21]	1.52 [1.47–1.57]	1.69 [1.56–1.82]	2.05 [1.93–2.17]	1.45 [1.33–1.59]	1.35 [1.27–1.44]
Painkillers use frequency	1.52 [1.46–1.57]	1.52 [1.47–1.57]	2.05 [1.93–2.17]	2.05 [1.93–2.17]	1.35 [1.27–1.43]	1.35 [1.27–1.44]
**Health risk behaviours**						
Fruit consumption	0.71 [0.68–0.73]	0.81 [0.78–0.83]	0.68 [0.63–0.73]	0.82 [0.76–0.88]	0.96 [0.89–1.04]	1.01 [0.94–1.09]
Physical activity	0.84 [0.82–0.86]	0.86 [0.84–0.88]	0.77 [0.74–0.82]	0.84 [0.80–0.89]	0.92 [0.87–0.97]	0.98 [0.93–1.04]
**COVID-19 related problems**						
Mental health	1.30 [1.23–1.36]	1.21 [1.15–1.28]	1.65 [1.51–1.80]	1.63 [1.48–1.80]	1.27 [1.15–1.41]	1.34 [1.21–1.49]
Relationship with parents	1.37 [1.30–1.45]	1.25 [1.18–1.33]	2.36 [2.15–2.58]	1.95 [1.76–2.16]	1.72 [1.55–1.91]	1.55 [1.38–1.74]
Relationship with friends	0.93 [0.92–1.01]	0.92 [0.88–0.97]	1.24 [1.13–1.35]	1.22 [1.12–1.33]	1.28 [1.16–1.42]	1.33 [1.20–1.46]

OR = odds ratio. AOR = adjusted odds ratio controlled for age, gender, family affluence, parental education, parental control, and parent's permissiveness with adolescents' alcohol use. The OR can be interpreted as the change in odds to be member of a specific profile compared to a reference profile with one unit change in the correlate.

a Reference category.

Adolescents who reported lower parental control and higher parental permissiveness towards adolescent alcohol use were more likely than other adolescents to be in the polysubstance use profile compared to the other two profiles. Moreover, those in the polysubstance use profile were more likely to be offered cannabis in the previous 12 months and reported a higher incidence of conduct problems compared to the other two profiles. Regarding mental and physical health problems, adolescents with polysubstance use reported more depressive symptoms, higher levels of loneliness, a higher number of clinical psychologist visits, and a greater frequency of headaches, other pains, and painkiller use compared to the other two profiles.

Regarding health behaviours, adolescents who consumed lower quantities of fruit per week and reported a lower frequency of physical activity were more likely to be in the polysubstance use profile when comparing them to the non-user profile. Moreover, adolescents in the polysubstance use profile reported a higher number of COVID-19-related problems in relation to mental health and their relationships with parents and friends.

Compared with non-users, adolescents with the snus and alcohol profile showed a similar pattern of associations with correlates (but lower ORs) than when non-users were compared with adolescents in the polysubstance use profile.

## Discussion

We used LPA to classify patterns of substance use among a large nationwide sample of Norwegian adolescents in 2021 (N = 97,429). A three-profile model described the data best, with 91% identified as non-users, whereas 7% were defined as adolescents who mainly used snus and alcohol and 2% as adolescents with polysubstance use. Our study is the first to identify substance use profiles during the COVID-19 pandemic in a Nordic country. Results indicate that the two substance use profiles were related to many variables in adolescents’ lives, including parental socio-economic status, social relationships, physical and mental health problems, health risk behaviours, and challenges associated with the COVID-19 pandemic.

We identified a similar number and distribution of profiles to previous studies^4^^,^^5^: the most common profile was non-consumption, followed by a profile with alcohol and snus use, whereas the polysubstance use profile had the smallest size. A four-profile solution is another common profile distribution found in the literature.^5^ For example, a large-scale study among Californian adolescents identified four profiles: non-use, alcohol experimentation, and mild and frequent polysubstance use.^11^ If our four-profile solution had been interpretable, it might have mirrored these results. However, since we also included non-combustible nicotine products, it is difficult to draw comparisons with previous results. In particular, our study underlines that non-combustible nicotine products are relevant in determining current substance use patterns among adolescents. Indeed, snus use was part of both substance use profiles, highlighting the key role snus use has gained in Nordic countries.^24^ Similar patterns of nicotine use are found in non-Nordic contexts where, instead of snus, e-cigarette use has become more frequent among adolescents than the use of combustible cigarettes.^6^^,^^10^ Our results suggest that snus and alcohol use are central in defining drug use profiles, even during the pandemic, demonstrating that prevention efforts aiming at limiting the use of alcohol and non-combustible nicotine products are warranted.

We also found that adolescents with polysubstance use mainly used alcohol, as well as cannabis and other illicit drugs. In a previous study using more than a decade-old dataset from Swedish adolescents, polysubstance use comprised alcohol, tobacco, drunkenness, and inhalants.^4^ Recent reports from Norway have documented an increase in adolescent cannabis use in the last few years.^25^ Although we cannot establish a direct relationship between our results and the increasing use of cannabis, study results indicate that cannabis use is an important part of polysubstance use among Nordic adolescents. This is even more relevant, considering that consumption of, growing, and selling cannabis in Norway are illegal.

When we analysed correlates, we found that adolescents who used several substances showed a pattern of increased risk in various life domains. In line with other studies, boys, older adolescents, and adolescents with lower parental socioeconomic status were more likely to be in the polysubstance use profile.^5^^,^^15^ Likewise, adolescents were more likely to be in the polysubstance use profile when they experienced lower levels of parental control, reported higher levels of mental health problems and conduct problems, and lived in a social environment that encouraged the consumption of drugs.^5^^,^^16^^,^^26^^,^^27^

We are the first to add correlates about specific COVID-19 problems among adolescents. Results showed that adolescents in the polysubstance user profile presented greater COVID-related mental health and social problems. Previous results among Norwegian adolescents indicated increases in depressive symptoms during the pandemic, while satisfaction with social relationships remained stable.^14^ However, our results suggest that adolescents who engage in polysubstance use were more vulnerable to experiencing greater mental health and interpersonal problems during the pandemic.

Additionally, our results indicate that those with a polysubstance use profile experienced more somatic pain and used more painkillers than other adolescents, which is in line with previous studies.^4^ Finally, we examined correlates that are not common in polysubstance use studies among adolescents. Indeed, our study builds upon previous studies that found low vegetable intake^17^ and low physical activity^18^ to be related to alcohol and tobacco consumption. In this sense, our study provides novel insights, showing that polysubstance use is related to other health risk behaviours that might affect the physical health of adolescents.

The present study might inform future prevention and intervention efforts that aim to reduce substance use and associated health risk factors among adolescents. Universal school-based interventions are one of the most popular and effective intervention formats to reduce drug use among adolescents.^28^ Nonetheless, several interventions showed small or no effects, partly because they usually did not address multiple drug use.^28^ Our study suggests that adolescents who consume several drugs have a pattern of increased risk in a variety of life domains. Thus, it would be of interest to implement interventions in school settings that provide adolescents with the skills necessary to resist polysubstance use.^28^ For example, enhancing motivational, social, self-control, coping, and decision-making skills should be components of prevention programmes, as they have proven to have positive long-term impact on lowering polysubstance use among adolescents at risk.^29^

The present study has some limitations. First, we were unable to establish the directionality between the correlates and polysubstance use profiles because our data are correlational. Second, our data should be interpreted within a Nordic context where snus use among adolescents is more prevalent than in other countries whereas e-cigarette use is less prevalent. Thus, generalising our results across countries and cultures should be done with caution. Third, the indicator ‘other illicit drugs use’ did not differentiate between different substances, even though this category potentially consists of many drugs. Future studies should provide more detailed assessments of illicit drug consumption and examine associated risk factors, particularly in older adolescents, among whom illicit drug use is most prevalent. Fourth, substance use was assessed by self-reports in schools. Even though participating schools were instructed to conduct data collections as they would have conducted school examinations to avoid answers to sensitive questions being visible to others, some study participants may have refrained from reporting excessive drug use in the context of assessments at school. Fifth, substance use was assessed differently across types of substances in terms of the time frame of use (i.e., recent use, weekly use, or time frames not clearly specified). Thus, the present study does not provide conclusive information about whether adolescents with a polysubstance use profile consumed several types of drugs concurrently or whether different drugs were used on different occasions. Sixth, although our results showed a similar latent structure when we treated substance use indicators as categorical variables, we used continuous indicators to analyse the different variance-covariance structure of our data, which help to better define the underlying relationship between the drug use indicators under the same latent structure.^12^ Seventh, we assessed substance use among 13–18-year-olds, even though recent reviews propose adolescence to range from about age 10–24 years.^30^ Future studies should therefore extend the age frame for research on polysubstance use, including somewhat older age groups. Finally, data were collected only during the pandemic; thus, we were not able to identify the specific effects of the COVID-19 pandemic on adolescents' substance use. Future studies should replicate these results in the post-pandemic period.

In conclusion, our study addresses a gap in the literature by using a large nationally representative sample, including novel data about problems related to the COVID-19 pandemic and characterising newly emerging substance use profiles. In addition, we identified several psychosocial and health behaviour factors that correlate with polysubstance use and snus/alcohol use profiles compared to the non-use profile. Thus, our results serve to inform future prevention efforts and interventions that aim to reduce polysubstance use problems among adolescents.

## Contributors

Conceptualization: RRC, TvS, LCG; Data curation: RRC, TvS, AB; Formal Analysis: RRC, TvS, GK; Funding acquisition: TvS, AB; Investigation: RRC, LCG; Methodology: RRC, TvS, GK; Project administration: TvS, AB; Resources: TvS, AB; Software (licenced ownership): TvS; Supervision: TvS, LCG, GK; Validation: GK; Visualization: RRC; Writing – original draft: RRC, LCG; Writing – review & editing: RRC, GK, LCG, AB, TvS.

## Data sharing statement

Data are available for research, teaching and students through the Norwegian Agency for Shared Services in Education and Research (SIKT) at https://doi.org/10.18712/NSD-NSD3007-V3.

## Declaration of interests

None to declare.

## Appendix A Supplementary data

Traslated abstract to Norwegian

Supplementary tables (Table S1 and Table S2) and statistical code

## Footnotes

a Although the 4-profile model did not show a correct identification, it showed similar proportions for the *non-users* and *snus and alcohol use* profiles. However, the 4-profile model identified two polysubstance use profiles with similar mean distributions. It was difficult to analyse whether the separation between these two profiles was correct because of problems with estimating the variance of use of other illicit drugs.
