# Supplementary material for: Polysubstance use and its correlation with psychosocial and health risk behaviours among more than 95,000 Norwegian adolescents during the COVID-19 pandemic (January to May 2021): a latent profile analysis
Source: Lancet Reg Health Eur. 2023 Mar 9;28:100603. doi: 10.1016/j.lanepe.2023.100603 (PMC9996359; doi:10.1016/j.lanepe.2023.100603)
Supplement: Traslated abstract to Norwegian [file mmc1.docx]

**Polysubstance use and its correlation with psychosocial and health risk behaviours among more than 95,000 Norwegian adolescents during the COVID-19 pandemic (January to May 2021):**

**a latent profile analysis**

*This translation in Norwegian was submitted by the authors and we reproduce it as supplied. It has not been peer reviewed. Our editorial processes have only been applied to the original abstract in English, which should serve as reference for this manuscript.*

**Abstract in Norwegian**

**Bakgrunn**. Bruk av flere rusmidler utgjør en helserisiko blant ungdom, men vi vet lite om denne problemstillingen under COVID-19-pandemien. Målsettingen med studien er (i) å undersøke hva som kjennetegner profiler av rusmiddelbruk blant ungdom og (ii) å identifisere korrelater til slike profiler.

**Metode.** Nasjonale spørreskjemadata fra Norge i 2021 ble analysert ved hjelp av latent profilanalyse. Deltakerne var 97 429 ungdommer i alderen 13 til 18 år. Vi målte bruk av sigaretter, e-sigaretter, snus, alkohol, cannabis og andre ulovlige rusmidler. Psykososiale variabler, helserisikoatferd og COVID-19-relaterte problemer ble også målt.

**Resultater.** Vi identifiserte tre profiler: Ungdom som ikke bruker noen rusmidler (n = 88 890, 91%), ungdom som bruker snus og alkohol (n = 6 546, 7%) og ungdom som bruker flere rusmidler (flerbruksprofil; n = 1 993; 2%). Gutter, eldre ungdom, ungdom med lavere sosioøkonomisk status, ungdom som rapporterte lite foreldreoppsyn og høye nivåer av alkoholbruk blant foreldre og ungdom med mange psykiske helseproblemer, høye verdier på smerterelaterte variabler og mer helserisikoatferd hadde en høyere risiko for å være i flerbruksprofilen. Videre hadde ungdom med sosiale og psykiske helseproblemer knyttet til COVID-19 høyere risiko for å bruke flere rusmidler. Ungdom som bruker snus og alkohol viste lignende risikomønstre, men på et noe lavere nivå enn ungdom i flerbruksprofilen.

**Fortolkning.** Ungdom som bruker flere rusmidler har en mindre sunn livsstil, er mer utsatt for å oppleve psykososiale problemer og rapporterer flere problemer knyttet til COVID-19-pandemien. Forebyggende strategier for å redusere bruk av flere rusmidler kan bidra til å fremme livskvalitet hos ungdom på flere ulike livsområder.
