# Supplementary material for: Polysubstance use and its correlation with psychosocial and health risk behaviours among more than 95,000 Norwegian adolescents during the COVID-19 pandemic (January to May 2021): a latent profile analysis
Source: Lancet Reg Health Eur. 2023 Mar 9;28:100603. doi: 10.1016/j.lanepe.2023.100603 (PMC9996359; doi:10.1016/j.lanepe.2023.100603)
Supplement: Supplementary tables (Table S1 and Table S2) and statistical code [file mmc2.docx]

##### **SUPPLEMENTARY MATERIALS**

Table S1. Latent Profile Analysis (LPA) indicators on substance use and transformations used in the analyses

| **Type of drug use** | **Questionnaire items** | **Response options** | **%** | **Coding of variable** | **Total N** | **Missing** |
| --- | --- | --- | --- | --- | --- | --- |
| *Cigarette use* | *Do you smoke (cigarettes)?* | I have never smoked | 80 | 0 | 96,779 | 5,000 |
|  |  | I used to smoke, but I stopped completely now | 10 | 0 |  |  |
|  |  | I smoke less than once a week | 7 | 1 |  |  |
|  |  | I smoke every week, but not every day | 2 | 2 |  |  |
|  |  | I smoke every day | 1 | 3 |  |  |
| *Snus use* | *Do you use snus (tobacco that you put under your lip)?* | I have never used snus | 80 | 0 | 96,768 | 5,011 |
|  |  | I used to use *snus*, but I stopped completely now | 9 | 0 |  |  |
|  |  | I use *snus* less than once a week | 4 | 1 |  |  |
|  |  | I use *snus* every week, but not every day | 1 | 2 |  |  |
|  |  | I use *snus* every day | 6 | 3 |  |  |
| *E-cigarette use* | *Do you use e-cigarettes/vape?* | I have never used *e-cigarettes/vape* | 83 | 0 | 96,473 | 5,306 |
|  |  | I used to use *e-cigarettes/vape*, but I stopped completely now | 12 | 0 |  |  |
|  |  | I use *e-cigarettes/vape* less than once a week | 3 | 1 |  |  |
|  |  | I use *e-cigarettes/vape* every week, but not every day | 1 | 2 |  |  |
|  |  | I use *e-cigarettes/vape* every day | 1 | 3 |  |  |
| *Alcohol use* | *Have you ever drunk any kind of alcoholic drinks?* | Never | 43 | 0 | 96,642 | 5,137 |
|  |  | I have just tasted them a few times | 21 | 1 |  |  |
|  |  | Occasionally, but less than once a month | 19 | 2 |  |  |
|  |  | Quite regularly 1-3 times a month | 13 | 3 |  |  |
|  |  | Every week | 4 | 4 |  |  |
| *Cannabis use* | *How many times have you used hash or marijuana over the past year (past 12 months)?* | Never | 93 | 0 | 96,086 | 5,693 |
|  |  | Once | 2 | 1 |  |  |
|  |  | 2-5 times | 2 | 2 |  |  |
|  |  | 6-10 times | 1 | 3 |  |  |
|  |  | 11 or more times | 2 | 4 |  |  |
| *Use of other illicit substances* | *How many times have you used other drug (narcotics) over the past year (past 12 months)?* | Never | 97 | 0 | 96,016 | 5,763 |
|  |  | Once | 1 | 1 |  |  |
|  |  | 2-5 times | 1 | 2 |  |  |
|  |  | 6-10 times | 0.3 | 3 |  |  |
|  |  | 11 or more times | 0.7 | 4 |  |  |

Table S2. Descriptive statistics by latent substance use profile

| **Variable** | **N** | **Total**  **N = 97,429** | **Non-user**  **(n = 88,890)** | **Snus and alcohol use**  **(n = 6,546)** | **Polysubstance use**  **(n = 1,993)** |
| --- | --- | --- | --- | --- | --- |
| **Demographics** |  |  |  |  |  |
| Gender | 95,394 |  |  |  |  |
| Boys (*n*, %) |  | 47,108 (49%) | 42,096 (48%) | 3,821 (60%) | 1,191 (67%) |
| Girls (*n*, %) |  | 48,286 (51%) | 45,109 (52%) | 2,581 (40%) | 596 (33%) |
| Age (mean, *SD*) | 94,522 | 15.20 (1.61) | 15.10 (1.59) | 16.42 (1.25) | 15.82 (1.57) |
| **Socioeconomic status** |  |  |  |  |  |
| Family Affluence (mean, *SD*) | 97,397 | 2.48 (0.43) | 2.49 (0.42) | 2.41 (0.41) | 2.22 (0.71) |
| Parental education (mean, *SD*) | 97,428 | 1.86 (1.19) | 1.88 (1.18) | 1.58 (1.18) | 1.55 (1.23) |
| **Social and relational variables** |  |  |  |  |  |
| Parental control (mean, *SD*) | 96,448 | 2.11 (0.41) | 2.12 (0.39) | 2.02 (0.47) | 1.79 (0.73) |
| Parent’s permissiveness regarding offspring’s alcohol use | 96,726 |  |  |  |  |
| *No* (*n*, %) |  | 73,693 (76%) | 69,918 (79%) | 2,821 (44%) | 954 (49%) |
| *Yes* (*n*, %) |  | 23,033 (24%) | 18,367 (21%) | 3,657 (56%) | 1,009 (51%) |
| Having been offered cannabis the previous 12 months (mean, *SD*) | 96,427 | 0.30 (0.64) | 0.22 (0.55) | 0.94 (0.90) | 1.66 (0.68) |
| **Mental Health** |  |  |  |  |  |
| Depressive symptoms (mean, *SD*) | 95,097 | 2.14 (0.81) | 2.11 (0.80) | 2.34 (0.84) | 2.74 (0.94) |
| Loneliness (mean, *SD*) | 94,375 | 1.93 (1.02) | 1.92 (1.01) | 1.98 (1.04) | 2.48 (1.21) |
| Contacts with clinical psychologist (mean, *SD*) | 94,411 | 1.23 (0.73) | 1.21 (0.69) | 1.38 (0.91) | 1.77 (1.22) |
| **Conduct problems** (mean, *SD*) | 96,943 | 1.37 (0.57) | 1.31 (0.48) | 1.82 (0.76) | 2.71 (1.20) |
| **Pain-related factors** |  |  |  |  |  |
| Headache frequency (mean, *SD*) | 83,920 | 2.17 (0.86) | 2.15 (0.86) | 2.31 (0.87) | 2.56 (1.01) |
| Other pain frequency^a^ (mean, *SD*) | 86,056 | 2.31 (0.87) | 2.29 (0.86) | 2.40 (0.92) | 2.68 (1.02) |
| Painkillers use frequency^b^ (mean, *SD*) | 95,643 | 1.85 (0.92) | 1.82 (0.89) | 2.12 (0.98) | 2.54 (1.34) |
| **Health risk behaviours** |  |  |  |  |  |
| Fruit consumption (mean, *SD*) | 94,502 | 1.11 (1.16) | 1.14 (1.17) | 0.72 (1.00) | 0.69 (1.03) |
| Frequency of physical activity (mean, *SD*) | 95,137 | 4.55 (1.25) | 4.58 (1.23) | 4.29 (1.30) | 4.15 (1.62) |
| **COVID-19 related problems** |  |  |  |  |  |
| Mental Health (mean, *SD*) | 90,163 | 2.25 (0.68) | 2.23 (0.68) | 2.35 (0.70) | 2.48 (0.73) |
| Relationship with parents (mean, *SD*) | 87,643 | 2.17 (0.64) | 2.15 (0.63) | 2.27 (0.64) | 2.50 (0.67) |
| Relationship with friends (mean, *SD*) | 88,117 | 2.07 (0.78) | 2.07 (0.78) | 2.04 (0.82) | 2.20 (0.94) |
| **Substance use indicators** |  |  |  |  |  |
| Cigarette use | 96,799 |  |  |  |  |
| *Non-use* (*n*, %) |  | 87,184 (90%) | 83,461 (94%) | 3,136 (48%) | 587 (30%) |
| *I smoke less than once a week* (*n*, %) |  | 6,721 (7.0%) | 4,002 (4.5%) | 2,201 (34%) | 518 (26%) |
| *I smoke every week, but not every day* (*n*, %) |  | 1,699 (1.8%) | 662 (0.7%) | 717 (11%) | 320 (16%) |
| *I smoke every day* (*n*, %) |  | 1,195 (1.2%) | 247 (0.3%) | 416 (6.4%) | 532 (27%) |
| Snus use | 96,768 |  |  |  |  |
| *Non-use* (*n*, %) |  | 85,582 (88%) | 85,008 (96%) | 0 (0%) | 574 (29%) |
| *I use snus less than once a week* (*n*, %) |  | 3,534 (4.0%) | 3,298 (3.7%) | 23 (0.4%) | 213 (11%) |
| *I use snus every week, but not every day* (*n*, %) |  | 1,558 (1.7%) | 0 (0%) | 1,390 (21%) | 168 (8.6%) |
| *I use snus every day* (*n*, %) |  | 6,094 (6.3%) | 0 (0%) | 5,088 (78%) | 1,006 (51%) |
| E-cigarette use | 96,473 |  |  |  |  |
| *Non-use* (*n*, %) |  | 92,142 (95%) | 85,844 (97%) | 5,191 (80%) | 1,107 (57%) |
| *I use e-cigarettes/vape less than once a week* (*n*, %) |  | 2,587 (3.0%) | 1,571 (1.8%) | 743 (12%) | 273 (14%) |
| *I use e-cigarettes/vape every week, but not every day* (*n*, %) |  | 743 (1.0%) | 402 (0.5%) | 226 (3.5%) | 115 (5.9%) |
| *I use e-cigarettes/vape every day* (*n*, %) |  | 1,001 (1.0%) | 256 (0.3%) | 293 (4.5%) | 452 (23%) |
| Alcohol use | 96,642 |  |  |  |  |
| *Never (n, %)* |  | 41,883 (43%) | 41,637 (47%) | 177 (2.7%) | 69 (3.5%) |
| *I have just tasted them a few times (n, %)* |  | 20,257 (21%) | 19,874 (23%) | 284 (4.4%) | 99 (5.1%) |
| *Occasionally, but less than once a month (n, %)* |  | 18,047 (19%) | 15,590 (18%) | 2,022 (31%) | 435 (22%) |
| *Quite regularly 1-3 times a month (n, %)* |  | 12,473 (13%) | 9,124 (10%) | 2,765 (43%) | 584 (30%) |
| *Every week (n, %)* |  | 3,982 (4.0%) | 1,965 (2.2%) | 1,248 (19%) | 769 (39%) |
| Cannabis use | 96,086 |  |  |  |  |
| *Never (n, %)* |  | 89,864 (94%) | 84,980 (97%) | 4,653 (73%) | 231 (12%) |
| *Once (n, %)* |  | 2,279 (2.2%) | 1,456 (1.7%) | 687 (11%) | 136 (7.0%) |
| *2-5 times (n, %)* |  | 1,918 (1.9%) | 937 (1.1%) | 653 (10%) | 328 (17%) |
| *6-10 times (n, %)* |  | 563 (0.5%) | 207 (0.2%) | 152 (2.4%) | 204 (11%) |
| *11 or more times (n, %)* |  | 1,462 (1.4%) | 219 (0.2%) | 243 (3.8%) | 1,000 (53%) |
| Use of other illicit substances | 96,016 |  |  |  |  |
| *Never (n, %)* |  | 92,713 (97%) | 86,759 (99%) | 5,954 (93%) | 0 (0%) |
| *Once (n, %)* |  | 1,264 (1.0%) | 837 (0.8%) | 427 (7%) | 0 (0%) |
| *2-5 times (n, %)* |  | 917 (1.0%) | 140 (0.2%) | 0 (0%) | 777 (41%) |
| *6-10 times (n, %)* |  | 288 (0.2%) | 0 (0%) | 0 (0%) | 288 (15%) |
| *11 or more times (n, %)* |  | 834 (0.8%) | 0 (0%) | 0 (0%) | 834 (44%) |

**Note.** ^a^Stomach-ache, joint, neck or muscle pain.; ^b^Paracetamol, ibux or similar.

## Statistical code. MPlus 8.5 code for the four variance-residual covariance structures

**Code common to all models**

VARIABLE:

NAMES = smoke snus ecig ah maria narco !substance use indicators

female age_clas FASmean parentuni !correlates

parcontro ah_prnts so_mari12

deprM lonely psychol conductP

headAche otherPain painkiller

fruit PA_freq

covidMH covidPe covidPa ID;

USEVARIABLES = smoke snus ecig ah maria narco;

MISSING = ALL (-999);

CLASSES = c (3);

!AUXILIARY = (R3STEP) female age_clas FASmean parentuni parcontro

!ah_prnts; !automatic three step method to test the relationship of !covariates with latent profiles.

**ANALYSIS:**

TYPE = mixture; ESTIMATOR = MLR;

PROCESSORS = 4; SITERATIONS = 20;

STARTS = 100 50;! We used for all models 100 50/ 200 100/ 1000 500.

! When models did not converge with these starting values

!we used 10000 5000.

!* data for TECH 11 and TECH 14

OPTSEED = 846194;

K-1STARTS = 10 5; !default # of initial iterations & # of final iterations;

LRTBOOTSTRAP = 1000; !number of bootstrap draws;

LRTSTARTS =10 5 100 50; !# of starts and optimizations for TECH14;

*!

**OUTPUT:**

TECH1 TECH8 TECH12 TECH13 TECH10 TECH11 TECH14

PATTERNS SVALUES RESIDUAL SAMPSTAT STDYX CINTERVAL

**!Models**

!!Each of the following sections run a distinct model to test !!the different variance covariance structures.

**!***

1. **Profile-invariant diagonal*!**

**MODEL:**

%OVERALL%

[smoke sus ecig ah maria narco]; !means

smoke snus ecig ah maria narco (var1-var6); !variances

!residuals correlations set to 0

smoke with snus@0; smoke with ecig@0; smoke with ah@0;

smoke with maria@0; smoke with narco@0; snus with ecig@0;

snus with ah@0; snus with maria@0; snus with narco@0;

ecig with ah@0; ecig with maria@0; ecig with narco@0;

ah with maria@0; ah with narco@0; maria with narco@0;

%c#1%

[smoke sus ecig ah maria narco]; !means

smoke snus ecig ah maria narco (var1-var6); !variances

!residuals correlations set to 0

smoke with snus@0; smoke with ecig@0; smoke with ah@0;

smoke with maria@0; smoke with narco@0; snus with ecig@0;

snus with ah@0; snus with maria@0; snus with narco@0;

ecig with ah@0; ecig with maria@0; ecig with narco@0;

ah with maria@0; ah with narco@0; maria with narco@0;

%c#2%

[smoke sus ecig ah maria narco]; !means

smoke snus ecig ah maria narco (var1-var6); !variances

!residuals correlations set to 0

smoke with snus@0; smoke with ecig@0; smoke with ah@0;

smoke with maria@0; smoke with narco@0; snus with ecig@0;

snus with ah@0; snus with maria@0; snus with narco@0;

ecig with ah@0; ecig with maria@0; ecig with narco@0;

ah with maria@0; ah with narco@0; maria with narco@0;

%c#3%

[smoke sus ecig ah maria narco]; !means

smoke snus ecig ah maria narco (var1-var6); !variances

!residuals correlations set to 0

smoke with snus@0; smoke with ecig@0; smoke with ah@0;

smoke with maria@0; smoke with narco@0; snus with ecig@0;

snus with ah@0; snus with maria@0; snus with narco@0;

ecig with ah@0; ecig with maria@0; ecig with narco@0;

ah with maria@0; ah with narco@0; maria with narco@0;

**!***

1. **Pofile-varying diagonal*!**

**MODEL:**

%OVERALL%

[smoke sus ecig ah maria narco]; !means

smoke snus ecig ah maria narco (var1-var6); !variances

!residuals correlations set to 0

smoke with snus@0; smoke with ecig@0; smoke with ah@0;

smoke with maria@0; smoke with narco@0; snus with ecig@0;

snus with ah@0; snus with maria@0; snus with narco@0;

ecig with ah@0; ecig with maria@0; ecig with narco@0;

ah with maria@0; ah with narco@0; maria with narco@0;

%c#1%

[smoke sus ecig ah maria narco]; !means

smoke snus ecig ah maria narco (var1-var6); !variances different by class

!residuals correlations set to 0

smoke with snus@0; smoke with ecig@0; smoke with ah@0;

smoke with maria@0; smoke with narco@0; snus with ecig@0;

snus with ah@0; snus with maria@0; snus with narco@0;

ecig with ah@0; ecig with maria@0; ecig with narco@0;

ah with maria@0; ah with narco@0; maria with narco@0;

%c#2%

[smoke sus ecig ah maria narco]; !means

smoke snus ecig ah maria narco (var7-var12); !variances different by class

!residuals correlations set to 0

smoke with snus@0; smoke with ecig@0; smoke with ah@0;

smoke with maria@0; smoke with narco@0; snus with ecig@0;

snus with ah@0; snus with maria@0; snus with narco@0;

ecig with ah@0; ecig with maria@0; ecig with narco@0;

ah with maria@0; ah with narco@0; maria with narco@0;

%c#3%

[smoke sus ecig ah maria narco]; !means

smoke snus ecig ah maria narco (var13-var18); !variances different by class

!residuals correlations set to 0

smoke with snus@0; smoke with ecig@0; smoke with ah@0;

smoke with maria@0; smoke with narco@0; snus with ecig@0;

snus with ah@0; snus with maria@0; snus with narco@0;

ecig with ah@0; ecig with maria@0; ecig with narco@0;

ah with maria@0; ah with narco@0; maria with narco@0;

**!***

1. **Profile-invariant non-diagonal *!**

**MODEL:**

%OVERALL%

[smoke sus ecig ah maria narco]; !means

smoke snus ecig ah maria narco (var1-var6); !variances

!residuals correlations defined

smoke with snus@(COR1); smoke with ecig@(COR2); smoke with ah@(COR3);

smoke with maria@(COR4); smoke with narco@(COR5); snus with ecig@(COR6);

snus with ah@(COR7); snus with maria@(COR8); snus with narco@(COR9);

ecig with ah@(COR10); ecig with maria@(COR11); ecig with narco@(COR12);

ah with maria@(COR13); ah with narco@(COR14); maria with narco@(COR15);

%c#1%

[smoke sus ecig ah maria narco]; !means

smoke snus ecig ah maria narco (var1-var6); !variances

!residuals correlations defined

smoke with snus@(COR1); smoke with ecig@(COR2); smoke with ah@(COR3);

smoke with maria@(COR4); smoke with narco@(COR5); snus with ecig@(COR6);

snus with ah@(COR7); snus with maria@(COR8); snus with narco@(COR9);

ecig with ah@(COR10); ecig with maria@(COR11); ecig with narco@(COR12);

ah with maria@(COR13); ah with narco@(COR14); maria with narco@(COR15);

%c#2%

[smoke sus ecig ah maria narco]; !means

smoke snus ecig ah maria narco (var1-var6); !variances

!residuals correlations defined

smoke with snus@(COR1); smoke with ecig@(COR2); smoke with ah@(COR3);

smoke with maria@(COR4); smoke with narco@(COR5); snus with ecig@(COR6);

snus with ah@(COR7); snus with maria@(COR8); snus with narco@(COR9);

ecig with ah@(COR10); ecig with maria@(COR11); ecig with narco@(COR12);

ah with maria@(COR13); ah with narco@(COR14); maria with narco@(COR15);

%c#3%

[smoke sus ecig ah maria narco]; !means

smoke snus ecig ah maria narco (var1-var6); !variances

!residuals correlations defined

smoke with snus@(COR1); smoke with ecig@(COR2); smoke with ah@(COR3);

smoke with maria@(COR4); smoke with narco@(COR5); snus with ecig@(COR6);

snus with ah@(COR7); snus with maria@(COR8); snus with narco@(COR9);

ecig with ah@(COR10); ecig with maria@(COR11); ecig with narco@(COR12);

ah with maria@(COR13); ah with narco@(COR14); maria with narco@(COR15);

**!***

1. **Profile-varying non-diagonal *!**

**MODEL:**

%OVERALL%

[smoke sus ecig ah maria narco]; !means

smoke snus ecig ah maria narco; !variances

!residuals correlations freely estimated

smoke with snus; smoke with ecig; smoke with ah;

smoke with maria; smoke with narco; snus with ecig;

snus with ah; snus with maria; snus with narco;

ecig with ah; ecig with maria; ecig with narco;

ah with maria; ah with narco; maria with narco;

%c#1%

[smoke sus ecig ah maria narco]; !means

smoke snus ecig ah maria narco; !variances

!residuals correlations freely estimated

smoke with snus; smoke with ecig; smoke with ah;

smoke with maria; smoke with narco; snus with ecig;

snus with ah; snus with maria; snus with narco;

ecig with ah; ecig with maria; ecig with narco;

ah with maria; ah with narco; maria with narco;

%c#2%

[smoke sus ecig ah maria narco]; !means

smoke snus ecig ah maria narco; !variances

!residuals correlations freely estimated

smoke with snus; smoke with ecig; smoke with ah;

smoke with maria; smoke with narco; snus with ecig;

snus with ah; snus with maria; snus with narco;

ecig with ah; ecig with maria; ecig with narco;

ah with maria; ah with narco; maria with narco;

%c#3%

[smoke sus ecig ah maria narco]; !means

smoke snus ecig ah maria narco; !variances

!residuals correlations freely estimated

smoke with snus; smoke with ecig; smoke with ah;

smoke with maria; smoke with narco; snus with ecig;

snus with ah; snus with maria; snus with narco;

ecig with ah; ecig with maria; ecig with narco;

ah with maria; ah with narco; maria with narco;
